# Considering flood risk in spatial development planning: A land use conflict analysis approach

**DOI:** 10.4102/jamba.v11i1.537

**Published:** 2019-04-10

**Authors:** Dirk P. Cilliers

**Affiliations:** 1 Unit for Environmental Sciences and Management, North-West University, Potchefstroom, South Africa

## Abstract

Flooding is the predominant natural hazard observed in Africa, and it often leads to damage to property and losses in human lives. To ensure that the detrimental effects of flooding are kept to a minimum, flood-prone areas should best be left undeveloped. Spatial development planning is a tool that can assist disaster risk managers in ensuring the aforementioned. This article proposes the use of a land use conflict analysis approach through which flood risk can be considered in spatial development planning in a proactive manner, specifically contributing to the flood risk management effort. A land use conflict analysis approach, relying on a variety of spatial analysis techniques, was used to identify areas that were both suitable for residential development and free from flood risk in the Batlhaping Ba-Ga-Phuduhucwana tribal area in South Africa. It was found that only 8% of the study area met these criteria. A comparison between the identified 8% and the existing spatial development plan for the study area revealed that some of the areas portrayed as suitable for development in the current spatial development plan are in fact flood risk areas. The article illustrates the value that a land use conflict analysis approach might have for flood risk management when integrated with spatial development planning.

**Keywords:** flooding; land use conflict analysis; suitability analysis; flood-prone area; disaster management; spatial development planning; South Africa.

## Introduction

Flooding is a fairly widespread natural hazard that can often have severe adverse effects on humans as well as on natural and built environments (Ran & Nedovic-Budic 2016). According to the International Disaster Database (EM-DAT 2017), approximately 42% of what it terms ‘natural disasters’ in Africa and approximately 35% of natural disasters in South Africa were flood-related for the period 2000–2016, making it the biggest single natural disaster type on both the continent of Africa and in South Africa. The United Nations Office for Disaster Risk Reduction (UNISDR 2009) defines a ‘natural hazard’ as a ‘natural process or phenomenon that may cause loss of life, injury or other health impacts, property damage, loss of livelihoods and services, social and economic disruption, or environmental damage’. It further defines a ‘disaster risk’ as ‘the potential disaster losses, in lives, health status, livelihoods, assets, and services, which could occur to a particular community or a society over some specified future time period’ (UNISDR 2009). Put plainly, if people reside in an area that is known to be prone to seasonal or flash flooding (natural hazard) there is a definite disaster risk, as possible losses to lives and damage to property might ensue. Traditionally, disasters were viewed as the results of ‘acts of nature’ that could not be predicted and therefore not planned for (Van Niekerk 2006). Over the years this view has shifted, however, and individuals, communities and governments alike have made various attempts to minimise society’s exposure to known natural hazards (Coppola 2006). One approach that has gained popularity is disaster risk reduction (DRR), which can be defined as (ISDR 2004):

the conceptual framework of elements considered with the possibilities to minimise vulnerabilities and disaster risks throughout a society, to avoid (prevention) or to limit (mitigation and preparedness) the adverse impacts of hazards, within the broad context of sustainable development. (p. 17)

Disaster risk reduction can therefore ensure that the impact of natural hazards, and in this case flooding, are minimised or avoided altogether. One way through which DRR could be promoted is through spatial development planning, which according to Malele (2009) is aimed at, amongst other things, ensuring that people and development are protected from disasters and disaster risk. This view has been reiterated by numerous authors, such as Wamsler (2006), who calls for proactive and preventative development planning to ensure that post-disaster destruction is avoided, and Sutanta, Rajabifard and Bishop (2010), who argue that spatial development planning, as a forward planning tool, is a potentially valuable instrument for DRR as it allows for the strategic management of future land uses in such a way that known natural hazards are avoided. With regard to flood risk management, Tingsanchali (2012) points out that development planning is gaining popularity as a preventative approach to flood risk management. It can therefore be argued that approaches through which flood risk management could be integrated with spatial development planning should be investigated (Ran & Nedovic-Budic 2016). This article explores the use of a land use conflict analysis approach for achieving the aforementioned. The concept of land use conflict analysis is first introduced, followed by a brief reflection on DRR and spatial development planning in South Africa, the country where the proposed approach is applied in this study. The proposed approach, its application and the results are finally discussed.

### Land use conflict analysis

The concept of land use conflict analysis is not new and has been successfully applied in various research studies and across various contexts through the years. Land use conflict analysis is based on the premise of spatial multicriteria analysis, a technique that allows for the analysis of large quantities of spatial data representing a variety of features in the environment (Belton & Stewart 2002). The approach has been used in spatial development planning to evaluate conflicts between urban land uses (Dutta 2012a; Iojă et al. 2014; Onose et al. 2015), to explore land use dynamics (Dutta 2012b) and to develop urban growth scenarios (Cilliers & Drewes 2010). The approach has also been applied outside of spatial development planning to evaluate the susceptibility of high potential agricultural land to flooding (Rahman & Saha 2008), to assist in the delimitation of ecological corridors (Santos et al. 2018) and to assist in strategic conservation planning (Geneletti & Van Duren 2008). According to Iojă et al. (2014) the application of land use conflict analysis in spatial development planning has been found to be useful for improving the overall quality of planning instruments, increasing social awareness around conflicts and providing guidance on possible development constraints to prospective developers. Dutta (2012b) further adds that the approach has also shown value as a mechanism for the proactive management of the effects of urban sprawl. A well-known application of the approach is the land use conflict identification strategy (LUCIS) developed by Carr and Zwick (2005, 2007). Land use conflict identification strategy is an approach through which the conflict between three competing land uses – urban, conservation and agriculture – is evaluated. Areas most suitable to each of the three land uses are identified through a suitability analysis and then compared to identify and evaluate possible conflicts. Most studies – including those employing LUCIS – applied the land use conflict analysis approach mainly to investigate the conflicts between land uses, while very few studies incorporated the concept of disaster risk as part of the analysis, and even fewer considered flood risk specifically. One exception is a study by Pechanec et al. (2011) that compared flood risk areas to existing land uses as well as proposed land uses reflected in existing development plans. The study, however, did not employ a comprehensive land use conflict analysis. Considering flood risk analysis as part of the land use conflict analysis process could be achieved by integrating information on flood risk with a land use conflict analysis approach such as LUCIS.

### Disaster risk reduction and spatial development planning in South Africa

In the South African context, DRR was formalised through the promulgation of the *South African Disaster Management Act 57 of 2002*, which calls for DRR measures to be considered and implemented through various mechanisms and across all government sectors (Republic of South Africa [RSA] 2003). One such mechanism in which disaster risk should be considered is the integrated development plan (IDP) (RSA 2000, 2003; Van Niekerk 2006). Integrated development plans are forward planning tools that, according to South African law, must be developed by all local and district municipalities in South Africa (RSA 2000) and can be described as multisectoral ‘development plans’ that guide and inform, amongst many other things, land use management decision-making within a municipality (Retief & Cilliers 2017). Although municipalities are doing fairly well in the integration of disaster risk management into IDPs (Botha et al. 2011), a shortfall is that it is not always thoroughly considered in the spatial component of IDPs, the so-called spatial development framework (SDF). An SDF is similar to many spatial development planning tools used around the world and is essentially a map indicating where certain land uses or developments should be promoted or discouraged, that is, informing land use management. Although the guidelines for the development of SDFs clearly state that ‘disaster-prone areas’ should be reflected in all SDFs (DRDLR 2014), anecdotal evidence (through the informal review of ten municipal SDFs) suggests that this is not the case. Many SDF documents do not mention disaster management and disaster-prone areas are rarely shown on maps. The *South African Spatial Planning and Land Use Management Act* further stipulates that development should be promoted (in part through SDFs) in areas that are regarded as sustainable (RSA 2013). This can be understood as to say that development should be promoted in areas that are suitable in terms of socio-economic criteria, such as proximity to schools and jobs, as well as biophysical criteria, such as ecological sensitivity and flood risk. The spatial development planning process must therefore consider a broad range of issues, making it a complex and often challenging process. The approach proposed in this study could facilitate this task as it can assist in considering a variety of potential development opportunities and constraints.

### Study area

**FIGURE 1:**
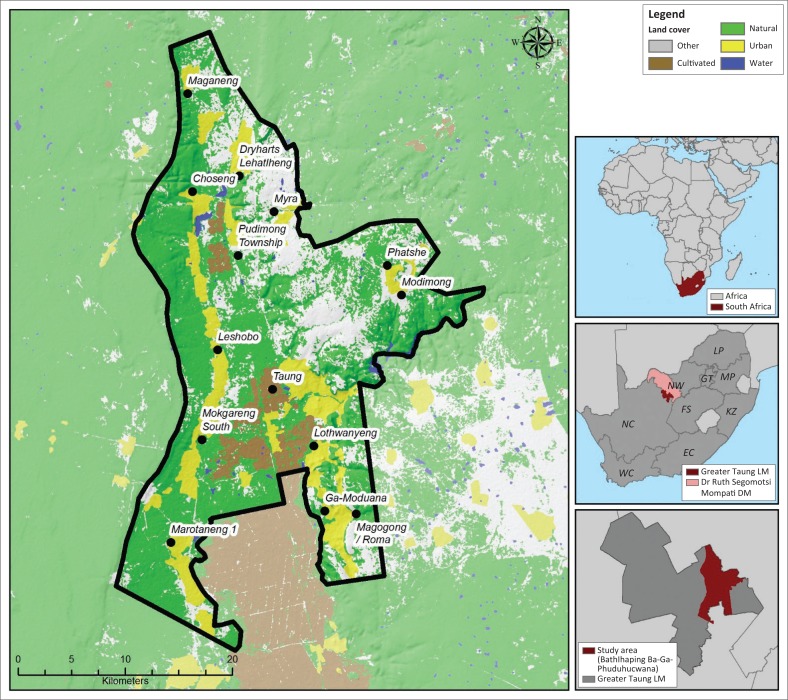
Study area.

The study area is approximately 853 km^2^ in size and covers most of the Batlhaping Ba-Ga-Phuduhucwana tribal area located in the eastern part of the Greater Taung Local Municipality, which falls within the North West Province of South Africa (Figure 1). The area was selected on the basis of the following considerations:

- The area has been affected by flooding in the past, resulting in loss of lives and damage to infrastructure.
- The area is faced with the challenge of people residing in flood risk areas.
- The area is located in a relatively poor and under-resourced (StatsSA 2011) rural municipality, which is representative of many rural municipalities in South Africa that are facing challenges with regard to disaster risk management.
- The area offered a variety of topography types ranging from flat plains to steep ridges and valleys.

The study area is jointly governed by the local municipality and the tribal authority, which often leads to challenges when dealing with, amongst other things, land use management (Ray, Quinlan & Sharma 2006). In the past, residences have often been developed impromptu and without following the necessary processes. The area is regarded as a rural area characterised by numerous small villages scattered along the Harts River Valley and has an average population density of between 50 and 200 people per square kilometre (StatsSA 2011). Less than 40% of the population is employed (StatsSA 2011), and those who are employed are mostly employed through social service programmes, the government sector or agriculture (CSIR 2011). Many of the dwellings in the area are categorised as informal dwellings (StatsSA 2011) and this, along with the fact that a large proportion of the population resides in close proximity to the rivers in the area, raises the likelihood that they might not be able to withstand the onslaughts brought about by floods, as was the case in recent flooding events. Although the area has a relatively low average rainfall of between only 400 mm and 600 mm per year, it has experienced extreme rainfall events more than once in the past 15 years. The most devastating event happened in 2006 when the area received approximately 1380 mm between January and June (Kabanda & Palamuleni 2013), which resulted in severe flooding on 28 March, killing six people, seriously injuring two children and leaving more than 1000 people homeless (Heslop 2008). Although the 2006 event was considered to be the worst in 18 years, less severe floods also occurred again in 2010 (26 January) and 2017 (23 February). The likelihood of seasonal flooding in the area coupled with the poor quality of some houses leaves the community vulnerable to flooding and emphasises the importance of guiding residential development towards areas that are both suitable for residential use and free from flood risk.

## Methodology

**FIGURE 2:**
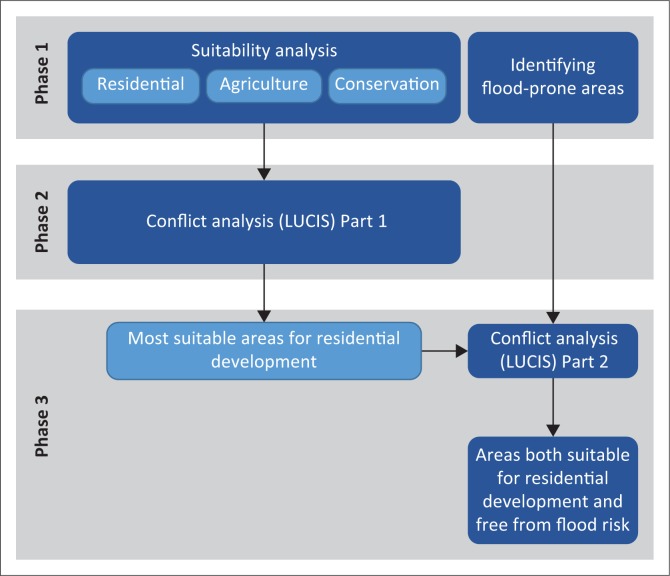
Analysis process. LUCIS, land use conflict identification strategy.

The methodology involved three phases as illustrated in Figure 2. The first phase entailed a suitability analysis to determine the most suitable areas for residential development, agricultural cultivation and biodiversity conservation. The selected land uses were adopted from the LUCIS approach, although the ‘urban’ class was refined to focus on residential development only, as the main concern was people residing in flood risk areas. It should be noted, however, that land uses can be adjusted to fit the context of a study area and are not limited to the three used in this study. Parallel to the three land uses, flood-prone areas were also identified. Phase 2 involved the application of the LUCIS approach through which areas most suitable for residential development were identified. In the final phase, the identified residential areas were analysed against the identified flood-prone areas to identify the areas that are most suitable for residential development and free from flood risk. The methodological approach will now be discussed in detail.

### Spatial data

A total of 13 datasets were used in the study. The datasets are listed in Table 1 along with a brief description of each dataset and the analysis that it was used for. All datasets were projected to the Universal Transverse Mercator Projection using Zone 35S using the WGS 1984 Datum. The data scale of datasets varied between 1:10 000 and 1:50 000, which means that the analysis results cannot be interpreted beyond a scale of 1:50 000.

**TABLE 1:** Spatial data.

Name	Description	Analysis
Distance from push factors	Euclidian distance between any location in the study area and the nearest push factor (industrial area, cemetery, landfill site or railway).	Residential suitability
Distance from residential areas	Euclidian distance from residential areas up to a maximum distance of 1 km. Classified into five classes using linear scaling; all areas further than 1 km are treated as a sixth class.	Residential suitability
Accessibility to key functions	Access to schools and clinics up to maximum walking time of 30 min. Classified into five classes using linear scaling; all areas greater than 30 min are treated as a sixth class.	Residential suitability
Distance from electrical infrastructure	Euclidian distance from electrical infrastructure up to a maximum distance of 1 km. Classified into five classes using linear scaling; all areas further than 1 km are treated as a sixth class.	Residential suitability
Slope (%)	Percentage slope rise classified into five classes up to a maximum rise of 15%. Classified into five classes using linear scaling; all areas with a rise of more than 15% treated as a sixth class.	Agriculture and residential suitability
Crop field boundaries	Depicts cultivated areas in eight classes ranging from fields that have not recently been cultivated to fields that are annually cultivated.	Agriculture suitability
Soil patterns	Depicts soil patterns in the area classified into four soil classes.	Agriculture suitability
Proximity to existing crop fields	Euclidian distance from cultivated areas up to a maximum distance of 1 km. Classified into five classes using linear scaling; all areas further than 1 km are treated as a sixth class.	Agriculture suitability
Prioritised features	Depicts the most important features for biodiversity conservation (protected areas, important bird areas, ridges, wetland clusters, critical biodiversity areas and ecological support areas).	Conservation priority
Likely riparian habitat	Depicts areas with characteristics associated with riparian habitat.	Conservation priority
Red data species habitat	Depicts habitat associated with listed species.	Conservation priority
Land cover	Depicts all natural and transformed areas in the study area (2014).	Agriculture suitability
Digital elevation model	Ten-metre resolution digital elevation model derived from elevation points and contours. Mean average error and root mean square error of 0.991 and 1.431, respectively.	Flood-prone areas

### Suitability analysis

Suitability analyses for the three competing land uses (see Figure 2) were conducted separately from one another. All datasets used during analysis were reclassified to a six-point scale ranging from zero to five, with zero representing criteria of least significance and five representing criteria of most significance. Saaty’s (1980) analytical hierarchy process was further used to allocate weights of importance to each of the datasets. The manner in which datasets were reclassified and the weights that were applied can be seen in Tables 2 through 4. The datasets for each component were analysed through a weighted overlay procedure in the ArcGIS ArcMap (version 10.5) software to determine suitability.

**TABLE 2:** Residential suitability.

Dataset†	Allocated values						Weight (AHP) %
	5	4	3	2	1	0	
Distance from push factors	> 250.0	150.10–250.00	100.10–150.00	50.00–100.00	< 50.00	N/A	20
Distance from residential areas	0.00–0.20	0.21–0.40	0.41–0.60	0.61–0.80	0.81–1.00	> 1	22
Accessibility to key functions	0.00–0.20	0.21–0.40	0.41–0.60	0.61–0.80	0.81–1.00	> 1	27
Distance from electrical infrastructure	0.00–0.20	0.21–0.40	0.41–0.60	0.61–0.80	0.81–1.00	> 1	13
Slope (%)	0.00–0.20	0.21–0.40	0.41–0.60	0.61–0.80	0.81–1.00	> 1	18

N/A, not applicable; AHP, analytical hierarchy process.

† , Also see Table 1.

**TABLE 3:** Agricultural suitability.

Dataset†	Allocated values						Weight (AHP) %
	5	4	3	2	1	0	
Crop field boundaries	Annual crops, horticulture, pivots, shade net and subsistence farming.	N/A	Old fields	N/A	N/A	Remainder	44
Slope (%)	0.00–0.20	0.21–0.40	0.41–0.60	0.61–0.80	0.81–10	> 1.00	14
Soil patterns	Very high potential soil	High potential soil	N/A	Low potential soil	N/A	N/A	13
Proximity to existing fields	0.00–0.20	0.21–0.40	0.41–0.60	0.61–0.80	0.81–1.00	> 1.00	29

N/A, not applicable; AHP, analytical hierarchy process.

† , Also see Table 1

**TABLE 4:** Conservation priority.

Dataset†	Allocated values						Weight (AHP) %
	5	4	3	2	1	0	
Prioritised features	Protected areas, important bird areas, ridges, wetland clusters and areas designated CBA-1	Areas designated CBA-2	Areas designated ESA-1	Areas designated ESA-2	N/A	N/A	55
Likely riparian habitat	Likely areas	N/A	N/A	N/A	N/A	Remainder	22
Red data species habitat	Likely areas	N/A	N/A	N/A	N/A	Remainder	23

Note: Land cover – used to remove transformed (non-natural) areas from the analysis result.

N/A, not applicable; AHP, analytical hierarchy process; CBA, critical biodiversity areas; ESA, ecological support areas.

† , Also see Table 1.

### Flood-prone areas

Flood-prone areas were identified using the height above the nearest drainage (HAND) procedure in TerraView 0.4.2. The HAND procedure identifies areas with high potential for flooding based only on the topographical characteristics and the drainage network of an area (Rosim et al. 2016), and therefore it does not employ streamflow, river cross section or rainfall data. Datasets such as the cross sections of rivers and high-resolution streamflow are rarely, if ever, available for rural municipalities in South Africa, which ruled out the use of most other flood models. It was important that a cost-effective approach relying on readily available data, and that could potentially be applied in all rural municipalities across South Africa, be selected for the identification of flood-prone areas – a requirement to which the HAND procedure conforms. A previous study that compared HAND flood results with an actual flood event showed the accuracy of the approach for the identification of areas with flood potential (Rosim et al. 2016). The HAND procedure requires a digital elevation model (DEM) as input, and the accuracy of the flood-prone areas identified through HAND is directly related to the quality of the DEM used. Elevation points and 5-m contours were used to generate a 10-m resolution DEM for the study area using the Topo to Raster tool in ArcMap version 10.5. Two accuracy tests were conducted to verify the accuracy of the DEM. The mean absolute error and root mean square error were 0.991 and 1.431, respectively, suggesting that the DEM was acceptable for further use in the analysis. Various thresholds ranging from 1000 to 300 000 were tested to determine a suitable drainage network in TerraView. A threshold of 15 000 was eventually used as it best represented the officially mapped 1:50 000 river network, which is widely used in South Africa and which also matched the scale of analysis. The HAND flood result was compared to a 100-year flood line that was available for a section of the study area.

### Conflict analysis (land use conflict identification strategy)

The LUCIS approach was applied across two phases (see Figure 2). It was first used to analyse the conflict between three key land uses in the area (residential development, agriculture and conservation) in an effort to determine the most suitable and conflict-free areas for residential development. The identified areas were then analysed against the flood-prone areas to identify the areas that were both suitable for development and free from possible flood risk. Map algebra was used to analyse the results for residential, agricultural and conservation suitability for conflicts. The three results were compared on a pixel-by-pixel basis where the suitability result with the highest score was selected. For example, if a pixel had residential, agricultural and conservation scores of respectively five, four and four, the final result would indicate the pixel most suitable for residential development. If the highest value was shared between two land uses, for example, if both residential and conservation scored values of five, the pixel was classified as a conflict pixel and not allocated to either. Only values of four and five were considered as they denote high suitability. Through this approach, only the pixels that were suitable for residential development and not in conflict with the two other two key land uses were identified. A similar approach was used to analyse the resultant suitable residential areas against the flood-prone areas to identify the pixels that were both suitable for development and free from flood risk. In a final step, the result was compared to the development proposals made by the existing SDF for the area.

## Results

**FIGURE 3:**
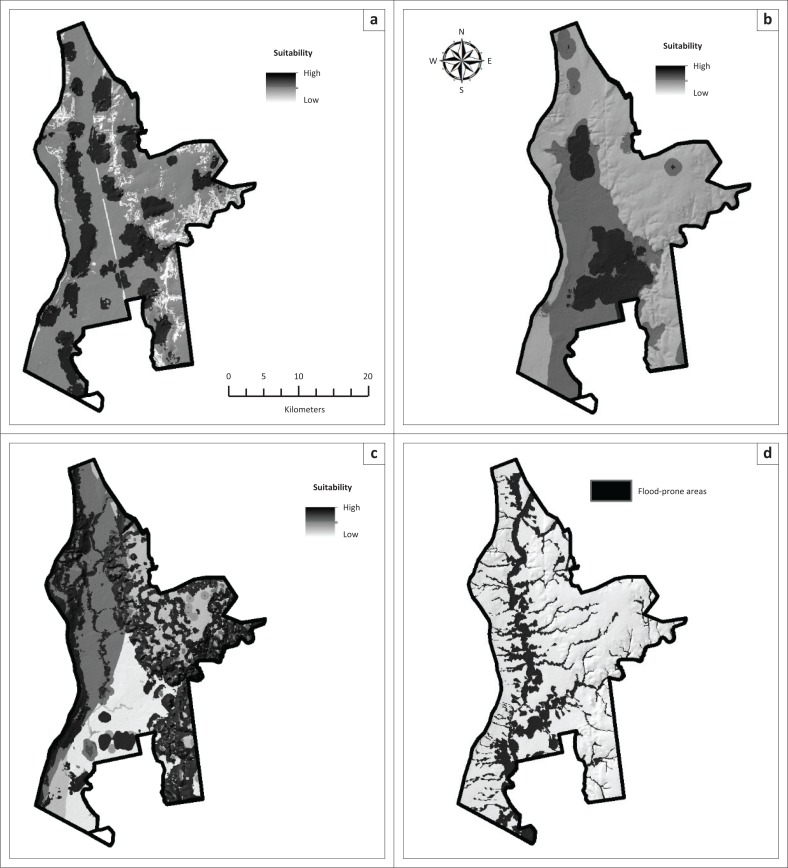
Suitability analysis results. (a) Urban, (b) agriculture, (c) conservation and (d) flood-prone areas.

The results for the suitability analysis are shown in Figure 3a, 3b and 3c. Areas of high suitability are shown in darker shades while areas of lower suitability are shown in lighter shades. Areas of high residential suitability are in close proximity to existing residential areas, which are scattered throughout the study area (see Figure 3a). The results show only three areas that are regarded as highly suitable for cultivation (see Figure 3b). This was expected as the study area is generally not considered as a high potential agriculture region. Areas important for biodiversity conservation are scattered all over the study area (see Figure 3c). The most sensitive features are along the ridges and rivers in the area. Figure 3d depicts the flood-prone areas identified through the HAND process. The most significant flood-prone areas straddle the Dry Harts (north to south) and the Harts Rivers (east to west), with smaller zones along their tributaries. The HAND procedure accurately predicted 84% of the flood-prone areas derived from the surveyed 100-year flood line, which was only available for part of the area, and was subsequently regarded as an acceptable representation of possible flood-prone areas for the whole study area.

**FIGURE 4:**
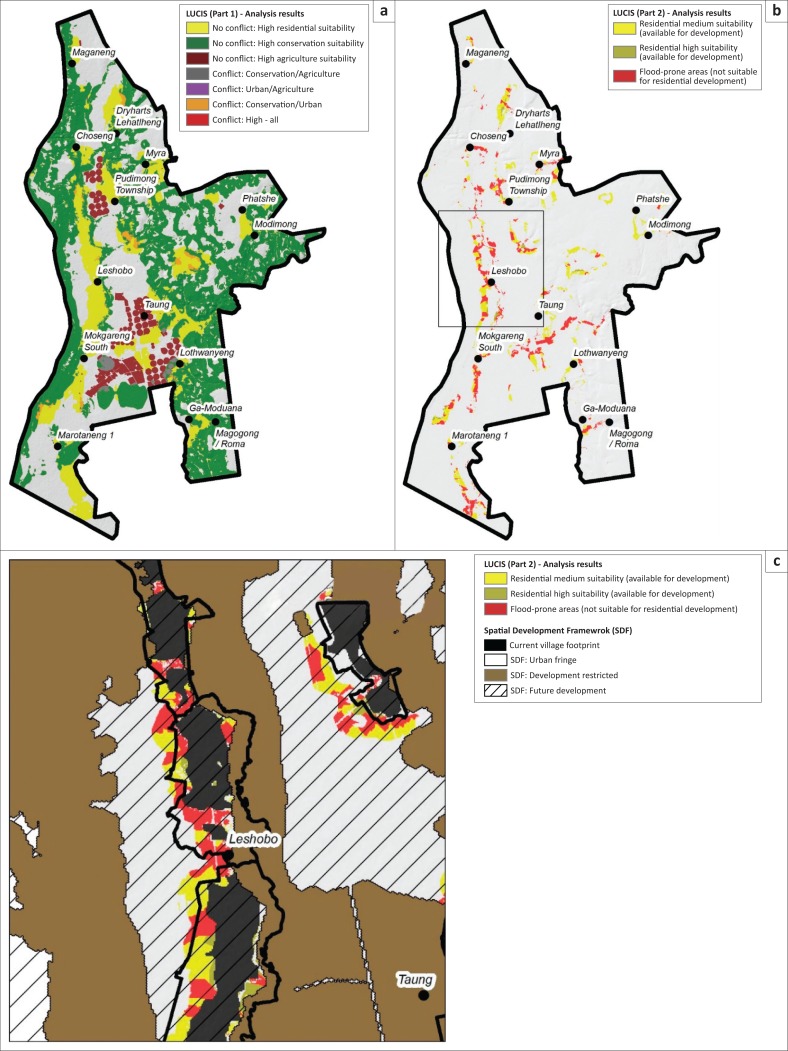
Conflict analysis results. (a) Conflict analysis, (b) conflict and risk-free residential development area and (c) Leshobo Village (extent shown on map). LUCIS, land use conflict identification strategy.

The results for the LUCIS analysis are shown in Figure 4. Figure 4a differentiates between areas of no conflict and areas of conflict. There were relatively few high suitable areas that were in conflict with one another (less than 2% of the study area) while a large proportion of the study area was not regarded as highly suitable (scores ≥ 4) for any of the investigated land uses (areas in grey – approximately 35% of study area). The largest portion of the study area, approximately 44%, was regarded as important for biodiversity conservation purposes. The final areas deemed suitable for residential development are shown in yellow and cover approximately 15% of the study area. Figure 4b shows the suitable residential areas when analysed against the flood-prone areas. The results show large portions of the identified suitable residential areas to be, in fact, unsuitable for residential development (areas in red). Figure 4c provides a more detailed view of one of the villages in the study area and is shown at a cartographic scale of 1:50 000. It shows the result when superimposed with the urban fringe, the future development zone and the development restriction zone, that is, the no-go area, as proposed in the current SDF. The comparison shows that some of the areas that have been earmarked for development in the SDF are in fact flood risk areas that should be avoided.

## Discussion

Although the current SDF did consider a partial 100-year flood line when earmarking areas for future development (Greater Taung Local Municipality 2015), it was not sufficient for identifying all possible flood risk areas, especially along the tributaries of the main rivers. The analysis shows the value that the proposed land use conflict analysis approach might have when considering flood risk, in a cost-effective manner, as part of the spatial development planning process. The following four learning points could be emphasised:

- A land use conflict analysis approach will ensure that optimal areas are allocated to each land use considered in the analysis, which will assist authorities in avoiding scenarios where people reside in areas that might be prone to flooding.
- Its proactive nature could help disaster managers and development planners to generate development scenarios, which could, in turn, be evaluated against flood risk scenarios. This should allow for more sustainable and resilient spatial development proposals and more informed disaster risk management strategies.
- Although development planning should consider applicable disaster risk management plans (in South Africa this is required by law for IDPs), the proposed approach will ensure that flood risk is also effectively considered in the relevant spatial analysis processes and not just in policy documents. This could mean that flood risk is considered at different levels of the planning and implementation process, which should result in more efficient DRR.
- The approach seems feasible for use in areas where datasets on streamflow and river cross sections are not available, although it should be said that the use of models that incorporate such datasets will further improve the results.

In the case of the study area, both the local municipality and the tribal authority could consult the proposed development scenario when making decisions on land use change (local municipality) or allocating portions of land to community members (tribal authority). As stated earlier, flooding is a widespread hazard that is expected to increase in intensity in the future because of factors such as climate change (Patz et al. 2005). It is therefore important that flood risk should be considered on as many levels as possible, and through as many mechanisms as possible, to ensure the safety and well-being of communities.

## Conclusion and recommendations

This study illustrates the value that a land use conflict analysis approach might have for flood risk management when integrated with spatial development planning. It should, however, be acknowledged that the success of the approach will be limited by the availability and quality of spatial data. In light of the aforementioned, it should also be noted that the HAND procedure, which is pivotal to the approach used in this study, requires only elevation data as an input – a dataset that is readily available for most countries is the world, including South Africa. This implies that flood-prone areas could be identified fairly easily for most areas (including rural areas) and that the approach presented in this article could therefore potentially be widely applied. Future research studies in which the approach is applied to regions with differing characteristics (urban centres, mountainous areas, mining regions, etc.) are needed to further improve and evaluate the approach.
